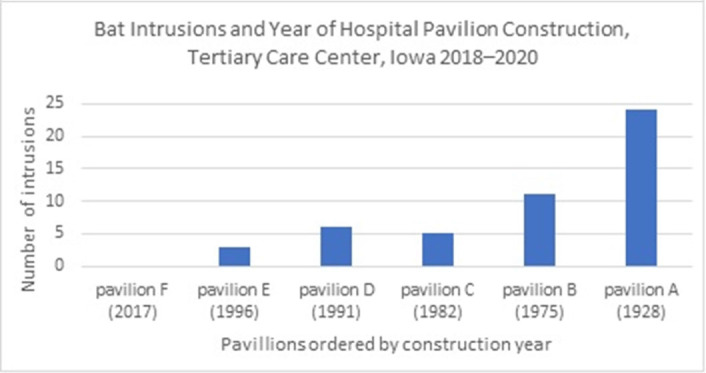# Bat Intrusions at a Tertiary Care Center, Iowa 2018–2020

**DOI:** 10.1017/ash.2021.29

**Published:** 2021-07-29

**Authors:** Mohammed Alsuhaibani, Takaaki Kobayashi, Lorinda Sheeler, Alexandra Trannel, Stephanie Holley, Oluchi Abosi, Kyle Jenn, Holly Meacham, William Etienne, Angie Dains, Mary Kukla, Bill Millard, Alexandre Marra, Melanie Wellington, Daniel Diekema, Jorge Salinas

## Abstract

**Background:** Bats are recognized as important vectors in disease transmission. Frequently, bats intrude into homes and buildings, increasing the risk to human health. We describe bat intrusions and exposure incidents in our hospital over a 3-year period. **Methods:** The University of Iowa Hospitals and Clinics (UIHC) is an 811-bed academic medical center in Iowa City, Iowa. Established in 1928, UIHC currently covers 209,031.84 m^2^ (~2,250,000 ft^2^) and contains 6 pavilions built between 1928 and 2017. We retrospectively obtained bat intrusion calls from the infection prevention and control program call database at UIHC during 2018–2020. We have also described the event management for intrusions potentially associated with patient exposures. **Results:** In total, 67 bat intrusions occurred during 2018–2020. The most frequent locations were hallways or lounges 28 (42%), nonclinical office spaces 19 (14%), and stairwells 8 (12%). Most bat intrusions (65%) occurred during the summer and fall (June–November). The number of events were 15 in 2018, 28 in 2019, and 24 in 2020. We observed that the number of intrusions increased with the age of each pavilion (Figure 1). Of 67 intrusions, 2 incidents (3%) were associated with potential exposure to patients. In the first incident, reported in 2019, the bat was captured in a patient care area and released before an investigation of exposures was completed and no rabies testing was available. Also, 10 patients were identified as having had potential exposure to the bat. Among them, 9 patients (90%) received rabies postexposure prophylaxis. In response to this serious event, we provided facility-wide education on our bat control policy, which includes the capture and safe handling of the bat, assessment of potential exposures, and potential need for rabies testing. We also implemented a bat exclusion project focused on the exterior of the oldest hospital buildings. The second event, 1 patient was identified to have potential exposure to the bat. The bat was captured, tested negative for rabies, no further action was needed. **Conclusions:** Bat intrusions can be an infection prevention and control challenge in facilities with older buildings. Hospitals may need animal intrusion surveillance systems, management protocols, and remediation efforts.

**Funding:** No

**Disclosures:** None

**Figure 1.**